# Topics of Public Interest

**Published:** 1917-07

**Authors:** 


### TOPICS OF PUBLIC INTEREST

**The English Recognize the Importance of Giving Authority to Medical Officers.** In 1904 the English War Office was reorganized by a committee, the chairman of which was Lord Esher. In this reorganization, no provision was made for a representative of the Medical Army Corps on the General Staff, or what corresponds to our War College. At the time the Surgeon General complained of this action. In reply to this complaint Lord Esher's committee stated that while too much importance could not be attached to the sanitary service of the army in peace or in war, the committee could not accept the views of the Surgeon General. Lord Esher's committee continued, "The Army Council is not, and can not be, a representative body as regards the several arms and departments. The Royal Army Medical Corps exists to serve the Army in a most important capacity, but the first object must be to create and maintain an Army, and this is the function of the Army Council. To admit the principle of representation would destroy the character of the Council."

This was the opinion of Lord Esher in 1904. Recently (London Times, February 3, 1917) Lord Esher writes as follows: "How much of the suffering undergone by our soldiers since the war began has been due to the shortsightedness of my committee, and notably of myself, will never be known. Certainly the control of the Adjutant General's branch over the Royal Army Medical Corps, was and is responsible not only for the early failure to grip the medical factors of the war, but they hampered conditions under which the Surgeon General has worked. His triumphs, and those of the Royal Army Medical Corps have been achieved in spite of obstacles that the subordination of science to ignorance, and of elasticity to military discipline explains, but can not justify." —V. C. V. The Journal of Laboratory and Clinical Medicine.

**Kultur.** We have called attention to the fact that while Kultur is etymologically identical with culture and preserves the figure of assisted growth, it implies the practical conception of the vegetable garden and of utilitarianism rather than the metaphor of the flower garden and the development of scholasticism. According to charges recently made, we may say that Kultur differs from culture as the rendering works differ from the crematory. The charge that the Germans are rendering fat and manure from human corpses depends entirely on the current meaning of the term Kadaver in Germany. The existence of rendering works in connection with the military organization and utilizing dead animal matter of some sort is plainly shown by German newspapers. Certain utterances of German officials, as that accredited to Admiral von Hintze that glycerin was being extracted from dead soldiers and some circumstantial evidence as the bundling together of several corpses and the railroad communication from the war fronts to rendering works, apparently confirms the charge that the Germans are resorting to this extreme mode of efficiency. However, the majority of the Entente newspapers are inclined to allow the benefit of the doubt by conceding the German claim that Kadaver without specification means an animal body in German. While from the strictly utilitarian standpoint, there is much to be said in favor of even this extreme method, we should not be so practical as to ignore the fact that the strongest force in human life is sentiment. In fact, most previous wars have been won by psychic states rather than force of arms and, in the long run, exceptions to this rule become reduced to a minimum.

---

**The Buffalo Children's Hospital** graduated the following nurses, June 20: Misses Arlene Briggs, Alison Maxwell Hall, Elizabeth Ingleby, Janet Morrison, Anna Guitdear, Anna Max, Laura Chase, Jessie Sunderland; Mrs. Mabel Browning.

---

**The Total Registration** of men between 21 and 30, was 9,649,938; the preliminary estimate ten million. In Buffalo, 39% claimed exemption.

---

**The Canadian Army Medical Corps** contains 1800 surgeons.

---

**Value of Anti-Variolar Vaccination.** Of 378 cases of small pox occurring in Connecticut in 10 months from July 1, 1916, only 10 had been previously vaccinated. All these vaccinations dated back at least 15 years and 5 were 30 years old.

---

**Capital Punishment** (by hanging) has been abolished in Missouri, the 13th state which now has no death penalty.

---

**Vital Statistics of Indians.** For 1916, the total Indian population of the U. S. was 209,224. Births numbered 6,092; deaths 4,570. It will be noted that the birth and death rates were about 29 and 20 per 1000, as compared with about 35 and 16, respectively, for the general population. But it must not be forgotten that our death rate is artificially lowered by the immigration of fairly healthy adults, with a marked preponderance of males, and that the average birth rate is increased by the high rate among the recently imported part of the population. With this allowance, the rates are not unfavorable. The American aborigines, so far as can be determined by ethnologic study, bred slowly and suffered a marked infant mortality, largely accounted for by the absense of substitute for mother's milk. It is probable that the Indian population is as great as it ever was before the discovery of America and it is certainly increasing.

---

**Centenarians.** A. C. Geyser, Med. Rev. of Revs., quotes statistics to the effect that the U. S. has 40 centenarians in 100 million inhabitants and Bulgaria 3,330 in 4 million. We do not believe that the statistics are accurate either way. In 12 issues, July 1916-June 1917, we mentioned, from definite and probably quite accurate press and medical journal reports, giving names, residences and dates, 22 centenarians, 9 alive, 13 very recently deceased. It is not conceivable that we simply happened on reports of more than half of all the centenarians in the country. As to Bulgaria, it is well known that statistics as to extreme longevity will fallaciously vary directly according to illiteracy. Given a poor and uneducated individual or class or nation, and in no offensive sense, it must be admitted that Bulgaria is such a nation in comparison with the U. S., records and personal knowledge of exact dates are unreliable. Both men and women are physically and mentally, at comparatively early ages and after 30 or 40 years of apparent senility, are commonly regarded as very old, even as centenarians, when their real age is somewhere between 75 and 95. In the hospital in which we served as interne, there was an old woman commonly called "Grossmutter" who quite sincerely believed herself to be a centenarian but had no exact knowledge of her age and who celebrated her birthday about twice a year. The U. S. Life Tables, based on the estimated population July 1, 1910, the reported deaths in 1909, 1910 and 1911, for registration states, give 50 native white males, and 80 native white females at ages from 100 to 106, per 100,000 born, i.e. of 100,000 born in any given year, these numbers will have survived. These tables are presumably as accurate as any that can be compiled. The population of the present area of the U. S. in 1815 was about 9 million. At the survival rates given above, there would be about 2,250 male and 3,600 female centenarians in the country at present, beside those representing survivals of immigrants. About half our population is composed of immigrants and their descendents but a considerable proportion of this half is of too recent advent to be included in an estimate of centenarians.

**Civil Service Examinations.** The position of Anatomist in the Army Medical Museum is vacant, salary $1600. ''At least a collegiate degree,'' a knowledge of the anatomy of disease-bearing mosquitos, of bacteriology, pathology and pathologic histology, ability to make photomicrographs, to prepare and keep in order museum specimens, are among the requirements. Examination July 11.

Male physicians are also needed for various services, at salaries ranging from $4 a day for actual employment, or $40 a month for part time, to $150 a month. Some of the positions involve ocean travel and participation in scientific research and the present vacancies are quite widely distributed geopraphically. Examination July 10. Candidates should apply immediately to the Civil Service Commission, Washington, D. C., for form 1312 and announcements No. 1174 and 1200.

---

**Camps for Medical Reserve Corps** have been established at Ft. Riley, Kas., Ft. Benjamin Harrison, Ind. and Ft. Oglethorpe, Ga. 700 will be accommodated. The full course of training covers 3 months, the first devoted to drill as enlisted men, the second to book work and the third to field practice. If necessary, the last and even the second month's training will be omitted but the first is regarded as essential. The high importance attached to the training of medical officers as enlisted men will seem strange to many persons but it needs no explanation to physicians.

---

**Utilization of Garbage, Etc.** The Dept. of Agriculture is urging all cities to have their chemists analyse city waste and to provide for its utilization. The separation of garbage from glass, tin, wood, string, etc., is especially urged, with a view to using the former for feedstuff for animals, as is practiced in all German cities of more than 40,000. It is estimated that the garbage from 17 million Germans yields enough compressed, protein-rich cattle feed to produce 1½ to 2 million liters of milk daily. (By the way, note that we say foodstuffs for human beings and feedstuff for the lower animals). Most American cities which attempt to reclaim waste at all, render the fat and either throw away or sell as fertilizer the remaining organic matter. American garbage contains about 3% of fat, German about 1%. Buffalo has one of the model plants of the country for reclaiming all kinds of waste but, with the tendency to reduction of fat in garbage, the German method of using the entire organic waste for feedstuff is more economic. This method has been in use in Omaha for several years, large numbers of hogs being fed. The H. C. L. has already had an influence on municipal waste. Garbage is reported as being reduced to half the former average in Chicago and some other cities and paper, formerly not worth saving except on a large scale, is now reclaimed to a considerable degree by private families and by persons supporting various philanthropies, instead of becoming an asset for the municipality.

---

**Gasoline Production for 1917,** is estimated at 2½ billion gallons, of which ½ billion will be exported.

---

**Railroad Economics.** It is estimated that the complete assemblage of man's apparel costs for transportation to any average point, 7 cents. The average receipts for all roads are 7½ per ton of freight per mile and less than 2 cents per passenger per mile. It is estimated that an increase of 1 mill per mile on both would yield a satisfactory net profit. However, there are obvious reasons for putting the increase onto freight alone, as the burden is then widely distributed, whereas the cost of human transportation is individual and often occurs in an emergency.

---

**Tuberculosis Hospitals** are proposed for Kansas, at an initial cost of $300,000.

---

**Italian Women Physicians** are serving in war hospitals for the first time.

---

**Pure Food and Drug Activities.** 1,036 successful suits against fraudulent nostrums were prosecuted in 1916. The packing and shipment of oysters are now under government control and it is believed that the danger of typhoid from this source is practically eliminated.

---

**Base Hospital Units for France.** On May 1, it was officially announced that the six following units had either sailed or would do so shortly: No. 2, Presbyterian Hospital, N. Y.; No. 4, Lakeside Hospital, Cleveland; No. 5, Harvard Medical School of Boston; No. 10, Pennsylvania Hospital, Philadelphia; No. 12, Northwestern University, Evanston; No. 21, Washington University Hospital, St. Louis.

---

**Poliomyelitis.** It is estimated that about 52,000 cases require after care in N. Y. State.

---

**The Colorado Fee Splitting Bill** requires that attendants must either render separate bills or that the proposed division shall be clearly stated. Bona fide permanent partnerships, the employment and retention of fees for services of assistants are allowed but services rendered by more than one partner must be itemized. Special partnerships for particular cases are not allowed.

---

**German Casualties** up to May 1, 1917, are said to total 4,245,804. About 1,000,000 have been killed. 1,500,000 of the casualties consisted of slight wounds. So far as can be judged, the permanent depletion of strength is about 2,500,000 out of a total available military strength of 7,000,000 at the beginning of the war and an increment of about 1,500,000 from the recruits of 1915-17. Similar estimates for May 1, 1915, gave over 1,600,000 killed, nearly 500,000 prisoners and over 1,800,000 wounded.

---

**Electric Delineation of Viscera.** The enthusiastic preliminary reports of this method, which we have already described in abstract, have not been fulfilled and the British Medical Journal practically withdraws its sanction and implies that the method should be forgotten.

---

**The Eclectic Examining Board of Florida** was deposed by the governor Mch. 2, on account of questionable prectices. Some one ought to have sufficient command of English to frame a definition of Eclectis, for legislative purposes, based on therapeutics instead of ethics.

---

**The Merger of the University of Pennsylvania, Jefferson and the Medico-Chirurgical Schools** represents a combined capital of 4 million and 6 million more is required.

---

**License for Vivisection Denied.** Rutgers College, New Brunswick, N. J., has been denied a license on the ground that the state law regulating such licensure and said to have been drawn especially with regard to the Rockefeller Institute, allows the granting of licenses only to institutions for scientific research and not to colleges and academies.

---

**Grain Storage.** The present situation regarding the cereal supply of the world suggests that the public granaries mentioned in the Bible and general in ancient literature were very practical forms of the war chest. Quite aside from war disturbances, it would in the long run be better for both producers, consumers and intermediate manufacturers such as millers and bakers, though not for speculators, if a fairly uniform price could be maintained for staple grains. The government, by buying at any rate below a fixed price and selling at a fixed maximum could not only benefit the people but perhaps make a profit that would reduce taxes and that would compete with private business only when, on the one hand, some form of state aid to farmers would be desirable and, on the other hand, when speculation or crop failures would justify a similar indirect aid to the ultimate consumers. Grains keep remarkably well, with very moderate precautions; indeed they have germinated after thousands of years in tombs and Pompeian bread has been found at least eatable after nearly 2000 years. The problem of storage is not so impracticable as might seem. The Buffalo elevators have a capacity of about 25 million bushels—a year's supply for the entire population of the state outside of N. Y. City. Boats, in harbor during the winter, in Buffalo, store 14-27 million bushels.

**Opportunity for Fame.** The same old plan of co-operative fame, inclosing $15 and photograph, has again been rendered available, for Buffalo and vicinity. If supposedly shrewd business men can even contemplate the success of the plan, why argue that we cannot afford war?

**Medical Reserve Corps.** Information from Surgeon General.

a. Grade for original commissions is determined by the Advisory Board and depends upon age, professional qualifications and former military experience. A great majority of original conmissions are in the grade of 1st Lieutenant.

b. All officers are eligible for promotion up to the grade of Major after a reasonable period of active service. They are entitled to pension for disability.

c. The service will be for the period of the war. A few officers may be retained after the war if the exigencies of the service require, but not unless they express a desire to be so continued in the service.

d. Circular of information regarding equipment and clothing is on file at the office of the Buffalo Medical Journal.

e. An officer is not required to furnish any equipment in the way of books, typewriters, etc.

**New State Traffic Regulations.** The bill discussed last fall, has become a law, with few alterations. It applies to all parts of the state except N. Y. City and with common sense qualifications, to all vehicles. Some of the principal points are as follows: In meeting or passing a street car, a 7-foot interval must be left. This apparently means that automobiles need not stop for a standing street car, if the street is of proper width but Main, Broadway, Niagara and Fillmore are the only streets in Buffalo on which cars run, which allow a 7-foot interval. Drivers should realize, however, that this does not allow them to plow at full speed through a line of pedestrians trying to board a car and, that in a down town district, the traffic officer has probably stopped all traffic parallel with the car tracks so as to allow vehicles to cross. It should also be remembered that the authorities recognize many cases in which a law does not mean what it says. Except for orders from traffic police, each vehicle must recognize the right of way of a vehicle approaching from a cross street at the right. It occurs to us that this rule will produce confusion when, as often happens, vehicles approach from both directions on two intersecting streets but, on the whole, it is better than right of way determined by points of the compass as there is always a question as to diagonal streets and between the claims of a thoroughfare crossing a little traveled street which has the right of way according to the compass. Moreover, in strange places, one is often confused as to points of the compass. Slowly moving vehicles must keep near the curb. This is a good law but drivers of such vehicles will not obey it and the police seem disinclined to inforce it, though they may, now that it is formally on the statute book. Vehicles emerging from an alley, garage entrance, etc., must be slowly and carefully driven with due warnings. Warning must be given of a turn, sudden decrease of speed, stop, or start when the latter involves turning into the stream of traffic. Vehicles must not stand farther than 6 inches from the curb, measured from both front and back wheels (several fines have already been imposed on this account), nor within 10 feet of a hydrant or cross walk, nor in the zone where street cars receive and unload passengers, nor within 15 feet of the entrance of any public building. Street cars have right of way between intersecting streets and vehicles must get out of the tracks immediately on warning. This rule and one about slowly moving vehicles ought to suffice to keep horse drawn vehicles from the middle of the road. This is one of the greatest present causes of congestion and, indirectly, of accidents. Use of the streets and public places as repair shops is forbidden, except in emergency. Trucks must be loaded so as to prevent loud noise, any material projecting more than 4 feet to the front or rear must be marked by a red flag or lantern. Bicycles and motorcycles shall not carry passengers except on secure seats, with provision of foot rests and hand grips. Persons are forbidden from riding on the rear of a vehicle without consent of the driver and from climbing onto standing cars or sounding horns on them. Hand carts and sleds are not allowed on streets or sidewalks except under local permissive regulations. We question, however, whether the police or any one else will be unduly severe with children. Reckless driving and jay-walking in the business districts are forbidden. Cities are authorized to enact local ordinances, but regulations must be posted conspicuously on the streets to which they apply, and we understand that they must not be directly antagonistic to the state law.

---

**Centenarians.** Thomas Wardall of Seattle, born in 1815, is still living. Amos Remington died near Harrisville, Vt., June 7, aged 101. He used tobacco and liquor all his life. Moral omitted. Mrs. Easter Waller died near Laurel, Del., June 7, aged 105.

---

**Military Statistics of Population.** The registration for the draft has ranged from 57.9% of the estimate of males 21-31, for Oregon, to 104.7% for Illinois, averaging about 90% and totaling 9,250,000. Estimates of aliens (unnaturalized) from enemy countries: Germany, 2,349,000, males over 21, 136,000; Austria-Hungary 1,376,000 and 447,000; Turkey 188,000 and 93,000; Bulgaria, 11,000 and 8,000. The relatively small number of adult male, unnaturalized Germans is due to the very general naturalization of this race up to 1910, and the return of reservists at the beginning of the war.

---

**N. Y. State Laws. Optometry.** The present board is continued. The board shall consist of 5 persons, the term for each member being 5 years, members being required to have practiced optometry 5 years. Educational requirements include two years of high school, increased to four after Jan. 1, 1920, 3 years experience in an optometrist's office or graduation from an approved school of optometry. The usual exemptions for those already in practice, are reenacted. Each customer or person fitted with glasses shall receive a bill of purchase with the signature, address and number of certificate of registration or exemption of the optometrist, a specification of the lenses and the price charged. Optometrists shall not assume the title of doctor. Fees, penalties for violation of law, etc., are provided.

**Consolidated Health Districts** may adopt the estimate system provided elsewhere and may issue certificates of indebtedness pending available resources from taxation.

**A Commission for Assuring an Adequate Food Supply** is provided and given general powers.

**A Bureau of Physical Training** is provided, in connection with the Military Training Commission, the present bill dealing mainly with salaries and allowances.

**Play Grounds and Recreation Centers.** This law authorizes the acquisition and management of such places in cities of the second class and villages.

**Undertaking.** Examination and license are provided on the general terms applying to the practice of medicine or special cults.

**Hospital Development Commission.** This is to examine sites, make general arrangements for development of existing and future hospitals, to accommodate properly the insane and feeble minded.

---

**State Hospital at Raybrook, Free Patients.** One year's state citizenship is required for males, 5 years' residence for females not citizens. Provision is made for filling vacancies on application to local health officers.

---

**State Civil Service Commission Announcement.** Assistant Chemist, $1,200-1,500. Physiological Chemist, $1,500. Supervising Matron and Dietitian, $720 and maintenance, Training School for Girls, Hudson, and possibly other vacancies from same eligible list. Physical Instructor, female, $45 a month and maintenance, at Training School at Hudson, Western House of Refuge at Albion, Reformatory at Bedford, and, at lower salaries, at various state hospitals. Trained Nurse, male and female, $420-600 and maintenance, at various institutions. Applications should be made before July 16 to the State Civil Service Commission, Albany, for forms. Examinations will be held July 28.

---

**The National Committee for Mental Hygiene** has created a sub-committee on furnishing hospital units for nervous and mental disorders to the United States government, with the approval of the Surgeon General. Dr. Pearce Bailey of New York is chairman. Further information and application forms on application to the National Committee for Mental Hygiene, 50 Union Square, New York City.

---

**The American Red Cross** is one of the Government activities in the line of preparedness. During times of peace the Red Cross maintains a skeleton organization of all its varied activities, assuming the obligation to immediately expand in every direction so as to undertake the tremendously responsible position it has conferred on it by Act of Congress in time of war. The Regular Army, the National Guard of the different states, all make plans for the medical and surgical care of its soldiers on a strictly peace basis. No proper provision can be made under their organization in their medical departments for anything more than first-aid and field hospital care. An army must be kept as mobile as possible, so there is no arrangement made during peace times for the equipment and transportation of large massive hospitals capable of taking care of hundreds of badly wounded soldiers. In the words of the military department the army can only take care of its sick and wounded men in the first zone; that is near the firing line. The further care of injured men is assumed by the organizations of the American Red Cross. Anyone who knows anything of military affairs can see the necessity of maintaining some military control of the soldiers after they have passed out of the first zone and have been sent further back of the lines. The Red Cross, by act of Congress, is the only medical organization permitted to take this control. Recognizing the value of a modern well equipped general hospital, with its staff of specialists, its more cumbersome equipment, the army asks the American Red Cross to organize and equip a series of hospitals with their staffs to be placed as near the first line as safety allows, to take over the care of the badly injured soldier, giving them the same treatment they would receive if they were civilians and came to a modern civilian hospital. These hospitals are called ''Base Hospitals,''—a misnomer from a civilian point of view—but properly in a military sense, as they are at or near a supply base. On request of the Washington authorities the Buffalo General Hospital was asked to equip and supply the staff of one of these hospitals. To furnish the duplicate of a modern hospital in beds, furnishings, X-ray instruments, operating equipment, etc., it has been found to come nearer in cost to $100,000 than the $25,000 originally estimated by the Army medical officers. Buffalo has almost completed its first Base Hospital. Buffalo should begin at once to organize and equip another just as soon as this first one is ordered for duty. The total number of Base Hospitals under organization will give accommodation to 25,000 wounded and sick men. This is not enough if the United States places 2,000,000 men on the firing line.—(Statement prepared by Dr. Marshall Clinton.

**University of Buffalo Commencement.** June 8, exercises were held jointly by all departments, this being the 71st annual commencement for the Dept. of Medicine, the 30th for Pharmacy, 29th for Law, 25th for Dentistry, the Dept. of Arts and Sciences founded in 1913, not yet having a graduating class. 60 were graduated in medicine, two diplomas being withheld as the graduates were under 21. The list of graduates, with hospital appointments, is appended. Drs. Kujawa, Moscato, Patterson and Pelham graduated with honors.

The Arthur G. Bennett Prize in Ophthalmology was awarded severally to Francis M. Kujawa and Vincent Charles Moscato, both having the same standing. The Roswell Park Book Prize in Surgery was awarded severally to Francis Joseph Butlak and Franklin Austin Knope, both having the same standing. The silver skull presented by the instructors in Anatomy for the first two years was awarded to Frank H. Valone.

Atkins, Leslie J., Buffalo General Hospital.
Barone, Nathaniel L., German Deaconess Hospital, Buffalo.
Butlak, Francis J., Sisters' Hospital, Buffalo.
Campbell, Robert J., Moses Taylor Hospital, Lackawanna.
Davies, Gertrude R., Children's Hospital, Buffalo.
Derby, Eugene F., Oklahoma General Hospital.
DeVita, Michael A. R., Cincinnati General Hospital, Cincinnati, Ohio.
Dobbie, Robert Paul, Buffalo General Hospital.
Dominicis, Rocco N., German Deaconess, Buffalo.
Eaton, Earl L., Buffalo General Hospital.
Ellison, Albert R., Erie County Hospital, Buffalo.
Fischer, Urban A., Emergency Hospital, Buffalo.
Foley, Mary J., Women's Medical College Hospital, Philadelphia.
Gibson, Wm. J., Moses Taylor Hospital, Lackawanna.
Glover, Arthur C., Arnot-Ogden Hospital, Elmira.
Goundry, Thomas, Lafayette General Hospital, Buffalo.
Greco, Antony J., Buffalo General Hospital.
Griffin, Grace H., Rochester State Hospital, Rochester.
Hamilton, Samuel T., Emergency Hospital, Buffalo.
Hart, John G., Buffalo General Hospital.
Higgs, William E., Youngstown City Hospital, Youngstown.
Hoehman, Howard D., Emergency Hospital, Buffalo.
Johnson, Harold M., Cincinnati General Hospital, Cincinnati, Ohio.
Jones, Wm. H., Buffalo General Hospital.
Jung, Daniel, Jr., St. Luke's Hospital, Cleveland, Ohio.
Knope, Franklin A., St. Mary's Hospital, Rochester.
Kofford, Boyer S., Youngstown City Hospital, Youngstown.
Kujawa, Francis M., Sisters' Hospital, Buffalo.
Kummer, Clarence P., Buffalo General Hospital.
LaDuca, Joseph P., Emergency Hospital, Buffalo.
Laport, Raymond G., Emergency Hospital, Buffalo.
Luke, Ray H., Buffalo General Hospital.

Lojacono, Salvator C., Elizabeth Steele Magee Hospital,
MacNaughton, Wallace F., Boston Dispensary Hospital, Boston, Mass.
McClellen, Frederick, C., New York Post Graduate Hospital.
Moscato, Vincent C., Buffalo General Hospital.
Nowak, John J., New York Post Graduate Hospital.
Palmer, Frederick W., Youngstown City Hospital, Youngstown, Ohio.
Patterson, Harold A., follow Laboratory work.
Pelham, Kate, Detroit Hospital for Women.
Pfaff, Norman J., St. Mary's Hospital, Rochester.
Robinson, Francis J., St. Mary's Hospital, Rochester.
Schwartz, Frederick L., Erie County Hospital.
Scibetta, Samuel L., Columbus Hospital, Buffalo.
Scott, Harry A., Erie County Hospital.
Scully, Alice, New England Hospital for Women and Children, Boston.
Smith, Kenneth A., Moses Taylor Hospital, Lackawanna.
Strong, Lyman J., Buffalo General Hospital.
Swierat, John V., Kings Park State Hospital, Kings Park,L.I.
Tench, Ellsworth M., Cincinnati General Hospital, Cincinnati.
Teresi, Charles C., Erie County Hospital.
Thoma, Earl W., Buffalo General Hospital.
Thompson, Myron A., Buffalo General Hospital.
Tillou, Donald J., Arnot Ogden Hospital, Elmira, N. Y.
Traver, Haworth R., Panama Hospital, Panama.
Twist, Edward A., St. John's Hospital, Long Island.
Walsh, Anna P., New England Hospital for Women and Children, Boston.
Wood, Edwin F., Hahnemann Hospital, Rochester.
Yellen, Hiram S., German Deaconess Hospital, Buffalo.
Zielinski, Ladislau E., Sisters' Hospital, Buffalo.

---

**Localized Tetanus.** H. Roger, Le Progres Med., May 12, describes a case, strictly localized in one leg, death from purulent peritonitis due to enterococcus.

---

**Treatment of Peritonitis by Ozone.** O. Laurent, Le Progres Med., May 19, page 169, advocates this method supplemental to laparotomy, introducing the gas through the drainage tube.

